# Necrotizing enterocolitis: current understanding of the prevention and management

**DOI:** 10.1007/s00383-023-05619-3

**Published:** 2024-01-10

**Authors:** Xiaohan Hu, Hansi Liang, Fang Li, Rui Zhang, Yanbo Zhu, Xueping Zhu, Yunyun Xu

**Affiliations:** 1 Institute of Pediatric, Children’s Hospital of Soochow University, 92 Zhong Nan Street, Suzhou City, Jiangsu Province China; 2 Department of Neonatology, Children’s Hospital of Soochow University, 92 Zhong Nan Street, Suzhou City, Jiangsu Province China; 3 Jiangsu Key Laboratory of Gastrointestinal Tumor Immunology, The First Affiliated Hospital of Soochow University, Suzhou, Jiangsu Province China; 4 Department of Human Anatomy and Histology and Embryology, Soochow University, Suzhou, Jiangsu Province China; 5 Department of Oncology, The First Affiliated Hospital of Soochow University, Suzhou, Jiangsu Province China

**Keywords:** Neonatal necrotizing enterocolitis, Prevention strategy, Treatment, Susceptibility factors, Clinical presentation, Complication

## Abstract

Necrotizing enterocolitis (NEC) is one of the diseases in neonates, with a high morbidity and mortality rate, especially in preterm infants. This review aimed to briefly introduce the latest epidemiology, susceptibility factors, and clinical diagnosis and presentation of NEC. We also organized new prevention strategies by risk factors according to different pathogeneses and then discussed new treatment methods based on Bell's staging and complications, and the classification of mild to high severity based on clinical and imaging manifestations. Such a generalization will help clinicians and researchers to gain a deeper understanding of the disease and to conduct more targeted classification, grading prevention, and exploration. We focused on prevention and treatment of the early and suspected stages of NEC, including the discovery of novel biomarkers and drugs to control disease progression. At the same time, we discussed its clinical application, future development, and shortcomings.

## Introduction

Necrotizing enterocolitis (NEC) is a devastating and destructive intestinal necrosis syndrome of the immature intestine in newborns, especially in preterm and low birth weight infants (LBWIs), with a prevalence of about 1‰ [1], up to 11% in very low birth weight infants (VLBWIs) of < 1500 g [2], and up to 22% in extremely low birth weight infants (ELBWIs) of < 1000 g [3]. A systematic review by Jones and Hall provided the most recent data on the epidemiology of NEC [4]: the total mortality rate of infants diagnosed with NEC is 23.5%, with the highest mortality rate of 50.9% for ELBWIs with NEC combined with surgery. Even if they survive, their prognosis is poor, with neurodevelopmental disorders (NDD) and intestinal failure (IF) being the most serious complications, occurring in 24.8% and 15.2% of all children with NEC, and in 59.3% and 35.3% of children with NEC requiring surgery, respectively.

### Conventional understanding of NEC

The etiology of NEC has not been determined, however, prematurity is the main cause of the disease [5]. This is due to the fact that in preterm infants and in cases of intrauterine growth restriction, newborns have immature intestines with poor peristalsis, but high permeability and low secretion of gastric acid and digestive enzymes; thus, their viability is low [6, 7]. In addition, the intestinal mucosa is easily damaged and deteriorates under inflammatory conditions, leading to total necrosis and perforation, which in turn leads to systemic inflammation, causing complications, such as neurodevelopmental disorders and lung damage [8]. In addition, local ischemia and hypoxia and/or impaired microcirculation, disturbances in the intestinal flora, and formula feeding (NEC occurs mainly in preterm infants who have received enteral feeding) are also major pathogenic factors [6–8].

NEC can lead to intestinal inflammation and intestinal necrosis [9]. Currently, the confirmation of NEC diagnosis is complex and lacks clinical diagnostic indicators with good specificity and high sensitivity. A combination of the following clinical signs is generally required: sudden onset of feeding intolerance, abdominal distention, bloody stools, and signs of sepsis (i.e., changes in the heart rate, respiratory rate, temperature, and blood pressure) in preterm infants [10, 11]. Subsequently, the C-reactive protein (CRP) and procalcitonin (PCT) values in routine blood tests; acid replacement and coagulation in blood gas analysis and electrolytes; and blood, urine, fecal, and cerebrospinal fluid culture results in pathogenesis are taken into account. The current and commonly used clinical staging of NEC is the Bell scale, which integrates the clinical and radiological manifestations of the child and classifies NEC into stages I, II, and III according to severity [12]. In 1986, Kliegman and Walsh modified and refined the Bell scale and used it widely to grade the severity of the disease and to guide treatment [13].

In stage I (suspected stage), children exhibit mild intestinal symptoms, non-specific systemic symptoms, and radiographical changes. In stage II (confirmed stage), the child's systemic symptoms worsen, with obvious abdominal distension, abdominal pressure and abdominal wall edema, thrombocytopenia with metabolic acidosis, and typical pneumatosis of the intestinal wall on X-rays. The child is then treated with nasogastric decompression, intravenous fluids, and broad-spectrum antibiotics. In view of the medical treatment, Bell stages I and II are also collectively referred to as the "medical NEC" stage. In stage III (progressive stage), the child develops peritonitis and hypotension together with worsening of stage II symptoms, metabolic acidosis and shock in severe cases, pneumoperitoneum on imaging, multi-organ failure, and intestinal perforation in critically ill children [7]. For children with stage III NEC, it is clear that surgery is urgently needed [14], with 20–50% of these children undergoing surgery [15, 16]. A long length of the bowel is removed, which predisposes patients to short bowel syndrome (SBS). Children with SBS require long-term parenteral nutrition (PN) [14], increasing the risk of complications, including NDD, IF, abnormal development of intestinal structures, pulmonary sequelae, and cholestatic liver disease caused by an inflammatory cascade response [1, 17–20]. In addition, the surgery also increases NEC mortality from 3 to 30% [21].

### Hot topics in NEC

#### Development of new scores

Bell staging is the first criteria for necrotizing enterocolitis, and is widely used in clinical practice, so this standard is mainly mentioned in this paper. Despite wide use of Bell staging to define NEC, there are several limitations as discussed below. Bell staging is not an explicit case definition, this can lead to over- or underestimation of NEC as was reported in a recent Swedish cohort study [22]. In addition, neither Bell staging nor modified Bell staging accounts for baseline risk, particularly gestational age, which is a major risk factor that influences the baseline risk of NEC. In recent years, newer scoring criteria or diagnostic definitions for NEC have made certain new progress [23].

##### Vermont Oxford Network (VON) definition

VON is a collaborative, currently including more than 1200 hospitals around the world that supports benchmarking of outcomes and quality improvement. The VON criteria define NEC as a diagnosis at surgery or on post-mortem examination or based on clinical and radiographic criteria (comprises features from Bell staging). Recent reports have noted a declining incidence of NEC in the United States, from 7.1% in 2005 to 5.2% in 2014, using this definition [24].

##### Centers for Disease Control and Prevention (CDC) definition

The CDC is a US Health Agency that performs infectious disease surveillance through the National Health Safety Network. The CDC surveillance definition for NEC is similar to the VON definition, with some modifications. Surgical NEC is defined as meeting one of the following findings: surgical evidence of extensive bowel necrosis (> 2 cm of bowel affected) or surgical evidence of pneumatosis intestinalis with or without intestinal perforation [23].

##### Gestational age-specific case definition of NEC (UK)

The UK Neonatal Collaborative NEC (UKNC-NEC) Study Group developed a point-based gestational age-specific case definition using a population-based cohort of infants [25]. The authors reported a lower error rate in classifying infants with NEC when compared to the VON definition.

##### Two out of 3 rule

The 2 out of 3 rule (2 of 3) is a scoring system, and the authors who proposed this rule have highlighted defining NEC subsets based on possible etiology or risk factors [26].

##### Stanford NEC score

This Stanford NEC score was developed using a six-center cohort of 520 infants with suspicion of NEC [27], which can be used to classify the severity of disease and also determine the risk of progression of disease.

##### International Neonatal Consortium (INC) NEC workgroup definition

A workgroup of stakeholders was assembled by the INC to guide the development of a new definition of NEC [28]. The report recommends infants with NEC that do not meet the criteria for “preterm NEC” should be classified as either “atypical NEC” or “term NEC” for reporting in clinical research.

#### Clinical scenarios

The traditional "Textbook" NEC is less common in clinical practice and more common in atypical manifestations, such as staccato NEC and pan-intestinal NEC [29]. In view of the heterogeneity of clinical presentation of patients with NEC, Hackam et al. have recently described five different types of presentation [30]. This classification based on clinical scenarios can help clinicians better understand the pathogenesis and determine the best treatment [31].

##### “Textbook”NEC

“Textbook” NEC refers to the presentation in which a premature infant who is predominantly formula-fed develops abdominal distention and bloody stools, and which is associated with the presence of a characteristic finding on abdominal plain films termed pneumatosis intestinalis, which refers to the presence of gas within the wall of the bowel, including the reversibility of the accompanying septic process and the presence of comorbidities.

##### Persistent NEC without free air

This presentation refers to the infant who develops NEC as above (presence of pneumatosis intestinalis) and fails to improve clinically but does not demonstrate obvious intestinal perforation. In the absence of clear improvement, exploratory laparotomy may reveal patchy necrosis and evidence of acute or indolent intestinal perforations.

##### Portal venous gas and abdominal tenderness

This presentation refers to the child with abdominal findings of air within the portal system, which generally suggests significant intestinal necrosis in the setting of abdominal tenderness.

##### Staccato NEC

This presentation refers to the child with NEC who is initially relatively stable yet rapidly develops deterioration characterized by overwhelming sepsis accompanied by clinical and radiographic evidence of NEC that evolves over hours.

##### NEC totalis

The child with NEC totalis exhibits extensive necrosis that involves nearly all the small and large intestines. While NEC totalis may be suspected on abdominal X-rays based upon the extent of pneumatosis intestinalis, the diagnosis of NEC totalis is often only made at laparotomy.

Given the complexity of NEC diagnosis and the limited availability of treatments, basic and clinical research on NEC has favored early prevention and treatment to control the disease at the "medical NEC" stage, avoiding surgery in the Bell III stage, which can cause irreversible suffering to the child. To date, the most accepted prevention and treatment measures are standardized feeding regimens, progressive feeding, breast milk, prophylactic antibiotics, and probiotics [6]. In addition, with the development of NEC diagnosis and treatment research, some new prevention and treatment strategies are gradually emerging in the field of preclinical and clinical research. Therefore, this review analyzes the prevention and treatment protocols based on risk factors and mild to high severity grading of NEC, with the aim of developing individualized prevention and treatment protocols for children with NEC with different pathogeneses, thereby improving the cure rate and reducing complications in children with NEC.

## Prevention strategy of NEC

In recent years, researchers have explored new strategies to reduce the risk factors of NEC, and here, we classify them according to their predisposing factors and possible pathogenesis, and summarize the latest research results (Table 1).

**Table 1:** Prevention strategies for NEC susceptibility factors

Prevention strategy of NEC	
Susceptibility factors	Prevention strategies to reduce the risk factors (References)
Premature delivery and intestinal	Prophylactic use of prenatal glucocorticoids [32–34]
Immaturity	Guiding the mother's diet and antibiotic use [17, 35]
	Reducing obstetric complications in mothers [36]
Abnormal colonization of intestinal bacteria and the microbiota	Systematic use of probiotics [37, 38] but is controversial [39, 40]
Formula feeding and enteral nutrition	Breast milk [41–44]
	Donor breast milk [45, 46], but ethical issues need to be considered [47]
	Slow feeding patterns but risky [48]
Infection and inflammation	Interleukin 10 [51]
	Intra-intestinal strategies targeting TLR4 [52], such as amniotic fluid [53] and breast milk [54, 55]
	Chondroitin sulfate [56]
Local ischemia-hypoxia reperfusion	Direct peritoneal resuscitation [59, 60]
Injury of the intestine	PGE2 and its receptor PTGER4 [61]
	1.5% glucose intraperitoneal infusion [62]
	Reducing feeding during transfusion [63]
	Screening congenital heart disease [64]
	Supplementation with arginine [65, 66]
	Targeting HIF-1 and GLUT1 [67]
	Remote ischemic conditioning [68, 69]

### Prevention of premature delivery and intestinal immaturity

Prevention of preterm birth and promotion of intestinal maturation in preterm infants can help prevent the development of NEC. One study found that prophylactic use of prenatal glucocorticoids reduced the morbidity and mortality of premature delivery-related neonatal disorders, including NEC [32]. A meta-analysis of 15 randomized controlled trials showed that prenatal application of glucocorticosteroids was effective in reducing the incidence of NEC [33]. In addition, a review of 30 randomized controlled trials (including 8158 infants) showed that prenatal application of corticosteroids reduced the morbidity and mortality of preterm birth and NEC in newborns without significant harm to the mother or the newborn [34]. Similarly, guiding the mother's diet and antibiotic use, and intervening in her microbiota and infections, might also influence the early gut flora colonization pattern of the infant and prevent NEC [17, 35]. Evidence-based medicine has shown that currently, the most practical approaches to prevent NEC include optimizing maternal health care, reducing obstetric complications in mothers, and decreasing their risk of preterm birth [36].

### Prevention of abnormal colonization of intestinal bacteria and the microbiota

Early microbial ecological dysbiosis is associated with the occurrence of NEC, and therefore, research on NEC prevention has focused on the use of probiotics in past decades. Several meta-analyses reviewed clinical trials involving more than 10,000 infants and came to the overall conclusion that probiotics can prevent NEC [37]. Breast milk contains a large amount of probiotics; however, the amount of breast milk from mothers of preterm infants is clinically low, making the administration of probiotics a reasonable measure to prevent NEC. In addition, *bifidobacteria* and *lactobacilli* are equally effective in the prevention of NEC in children, although a mixture of *bifidobacteria* and *streptococci* is more effective; however, there is a lack of clinically accurate comparative data [38], and both the inclusion criteria and strain doses are major barriers to forming evidence-based conclusions [39]. Currently, the systematic use of probiotics for NEC prevention is controversial because of the complexity of the strains and the lack of valid evidence for their clinical use, in addition to a series of published cases showing that probiotics use can lead to adverse outcomes such as sepsis [40].

### Prevention of risks associated with formula feeding and enteral nutrition

Breast milk is rich in several bioactive factors that improve the gastrointestinal defenses of infants. Several clinical trials have shown that breastfeeding can standardize feeding regimens, prevent postnatal growth restriction, and reduce the incidence of NEC [41]. Two randomized trials have also demonstrated that breastfeeding reduces the incidence of NEC in preterm infants compared with formula feeding [42, 43]. A study involving 207 infants also found a significantly lower incidence of NEC in infants who were exclusively breastfed compared with those fed with breast milk fortified products [44].

Donor breast milk is also a good option when the mother is unable to provide breast milk. A systematic review and meta-analysis of 11 randomized or quasi-randomized trials, involving 1809 preterm or low birth weight (LBW) infants, found that donor breast milk was effective in preventing NEC [45], with a 64% reduction in the risk of NEC compared with breast milk fortification [46]. However, ethically, preterm infants should only receive pasteurized donor breast milk if they do not have their own mother's breast milk [47]. Notably, slow feeding patterns have been proposed; however, the available experimental data demonstrate that slow advancement of enteral nutrition might have little or no effect on the risk of NEC and mortality in preterm or VLBW infants, and might even slightly increase the risk of feeding intolerance and invasive infections [48].

Indeed, in a compelling article, “Can We Cut the Incidence of Necrotizing Enterocolitis in Half-Today?” [49], Dr Robert Christensen argued that adopting two practices: near-exclusive breast milk feeding and the use of standardized feeding protocols (SFPs), could do just that [50]. SFPs address a consistent approach to the: (a) preferred feeding substance; (b) advancement and fortification of feeding; (c) criteria to stop and specifying how to re-start feedings once held; (d) identification and handling of feeding intolerance; and (e) initiation and duration of trophic feeding. SFPs are simple, inexpensive, effective, and transmissible methods for prevention of NEC [41].

### Prevention of infection and inflammation

Prevention of infection and control of inflammation are equally crucial in the prevention and treatment of NEC. It was reported that interleukin 10 (IL-10) prevents disease progression in NEC mice by regulating intestinal inflammation [51]. Toll-like receptor 4 (TLR4) signaling plays a key role in mediating intestinal inflammatory imbalance, and intra-intestinal strategies targeting TLR4 in preterm infants might represent a new approach to NEC prevention [52]. Fetal intestinal lumen immersion in amniotic fluid has been documented to prevent TLR4 activation and thus NEC [53]. Breast milk is rich in the nitric oxide precursor, sodium nitrate [54], and oligosaccharide, both of which are absorbed in circulation and promote nitric oxide release [55]. This counteracts the effects of TLR4 signaling on the mesenteric endothelium, reducing intestinal inflammation, and thus decreasing the incidence of NEC. Recent studies have shown that chondroitin sulfate (CS) attenuates inflammation, prevents intestinal ecological dysregulation, and reduces the severity and mortality of NEC [56].

### Prevention of local ischemia-hypoxia reperfusion injury of the intestine

Studies have demonstrated the presence of microcirculatory injury in the intestine of children with NEC [57, 58], and this injury can be improved by direct peritoneal resuscitation [59, 60]. It was found that prostaglandin E2 (PGE2) and its receptor, prostaglandin E receptor 4 (PTGER4, also known as EP4) improved intestinal blood flow and provided intestinal protection against NEC in neonatal rats [61]. Similarly, 1.5% glucose solution delivered by intraperitoneal infusion enhanced ileal blood flow and prevented NEC in neonatal rats [62]. Several other clinical studies have shown that measures, such as reducing feeding during transfusion [63], screening children for congenital heart disease [64], and supplementation with arginine (arginine and/or citrulline, amino acids that play an important role in nitric oxide production and the regulation of intestinal blood flow, showing a reduced level in NEC) [65], can prevent NEC [66]. A recent study of intestinal tissue from children with NEC showed that the levels of hypoxia markers hypoxia-inducible factor 1 (HIF-1) and glucose transporter 1 (GLUT1) were elevated in NEC, suggesting their potential role in the prevention of reperfusion injury in NEC [67].

Remote ischemic conditioning (RIC) is a new approach to prevent and treat NEC. Preclinical studies have led to the discovery of remote ischemic conditioning (RIC) as a promising non-invasive intervention in protecting the intestine against ischemia-induced damage during early-stage NEC. RIC involves the administration of brief reversible cycles of ischemia and reperfusion in a limb (similar to taking standard blood pressure measurement) which activate endogenous protective signaling pathways that are conveyed to distant organs such as the intestine. RIC targets the intestinal microcirculation and by improving blood flow to the intestine, reduces the intestinal damage of experimental NEC and prolongs survival [68]. A phase II feasibility randomized controlled trial involving 12 centers in 6 countries is currently underway, to investigate the feasibility of RIC as a treatment for early-stage NEC in preterm neonates [69].

## Treatment of NEC

**Fig. 1:**
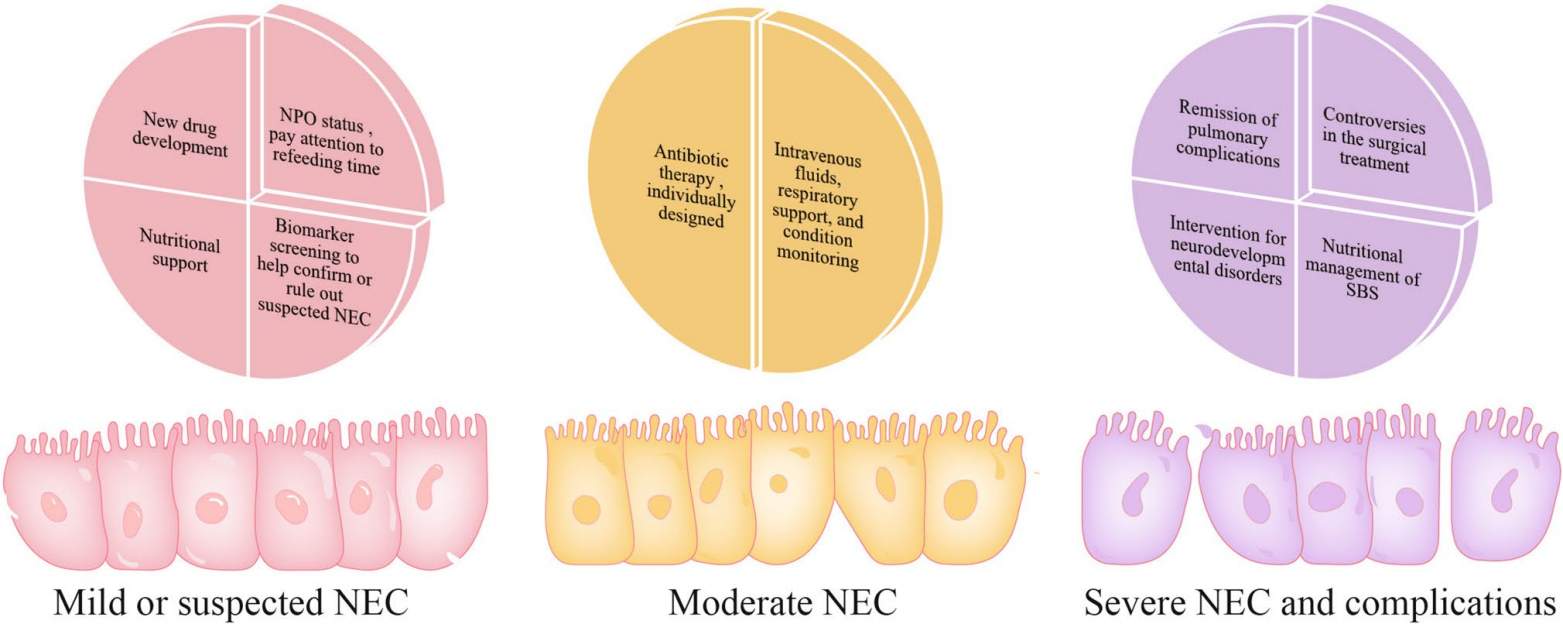
Summarized treatments for NEC according to disease severity

The early clinical manifestations of NEC are not specific, and the lack of specific biological markers often makes it difficult to diagnose. The most widely used is the Bell score, which integrates the clinical and imaging manifestations of the child. We will summarize the current treatments for NEC in a step-by-step manner based on the clinical manifestations and imaging examinations of the child in Bell's staging, and organize them according to disease severity (Fig. 1, Table 2).

**Table 2:** Treatments of NEC with different severity

Treatment of NEC	
NEC severity	Treatment (References)
Mild or suspected NEC	NPO status [70], pay attention to refeeding time [71, 72]
	Biomarker screening to help confirm or rule out suspected NEC [19, 80]
	Potential biomarkers
	Urine I-FABP [73]
	Intestinal I-FABP, claudin-3 [73] or inter-α inhibitor protein [74]
	Stool calprotectin [73, 75, 76]
	Ultrasound [77], NIRS [78] or microbiota [79]
	Multi-signal scoring systems
	ApoSAA [81]
	Integrated fecal calprotectin and urinary I-FABP [82]
	Urine rintso2 and intestinal I-FABP [83]
	Intestinal and stool IAP [84]
	Omics technologies [85]
	Metabolomics [86–89]
	Proteomics [90–95]
	Integrated metabolomics with either proteomics [96] or metagenomics [87, 97, 98]
	Nutritional support
	Enteral feeding time [99, 100]
	Breast milk [101, 102]
	Recognized nutrient components
	Mucosal integrity [104]: exosomes [110–112]
	Immune function [104]: IgA [105], Oligosaccharides [106], Exosomes [107–109]
	Donor breast milk [44, 113, 114]
	Purification of milk to obtain exosomes [118]
	PN: children undergoing NPO
	Intravenous nutrients [10, 119–121]; however, the role needs to be further confirmed [122]
	New drug development
	Novel biological agents (Need clinical trials)
	Heparin-conjugated EGF-like growth factor [57, 123–125]
	Oligosaccharide C34 [126]
	Breast milk oligosaccharides [127, 128]
	Amniotic fluid growth factors [53, 129, 130]
	Oral lactoferrin [131]
	Stem cells and their exosomes [132]
	New therapeutic pathways
	Silencing angiopoietin-2 to block the Notch signaling pathway [133]
	IL-1β [134]
	COX-2 inhibitor [135]
	Sodium butyrate [136]
	DHA [137]
	FFT [138]
	Amniotic fluid stem cell injection therapy [139]
	Tocilizumab inhibition of IL-6 [140]
Moderate NEC	Intravenous fluids, respiratory support, and condition monitoring
	Fluid restriction [142]
	Evaluating multiple organ function regularly [145]
	Respiratory support [146, 147]
	Antibiotic therapy, individually designed [151]
	No prospective or randomized studies of the safety or efficacy [148]
Severe NEC and complications	Controversies in Surgical treatment [152–159]
	Nutritional management of SBS
	Combination of PN and EN: stimulate the residual intestine while ensuring the energy needed [160]
	Intervention studies for neurodevelopmental disorders
	Targeting TLR4 signaling pathway [172]
	Remission of pulmonary complications
	TLR4 small molecule inhibitor C34 [174], targeting Th17 [176, 177], pre-digested fat system [178]

### Treatment of mild or suspected NEC

#### Suspension of enteral feeding strategy (Non-per os, NPO status)

Clinically, NPO significantly improves the early suspected symptoms of NEC; however, clinicians often hesitate about when to start refeeding after NPO because of the lack of an optimal time point for refeeding [70]. Similarly, a meta-analysis showed no significant difference in outcomes for children who resumed feeding early (within 5 days of NEC diagnosis) and late (> 5 days after NEC diagnosis) [71]. However, ultrasound was used to compare children who were fed without portal gas for 3 consecutive days with children who were fed without portal gas for 10 consecutive days, and it was found that early resumption of feeding had a lower complication rate, a shorter course of antibiotics, faster feeding progress, and a shorter hospital stay [72]. Therefore, the duration of NPO should be minimized clinically and the child should start to feed immediately after stabilization of vital signs, resolution of thrombocytopenia, and clinical improvement, as determined by abdominal radiography or ultrasonography.

#### Biomarker screening for the early identification of NEC

Unlike the sudden appearance of overt clinical signs in mid- to late-stage disease, the onset of NEC is often subtle and insidious; therefore, many researchers have attempted to identify new biomarkers from serum, stool, and urine samples to help confirm or rule out NEC [19]. Urine of children with NEC had significantly higher levels of intestinal fatty acid binding protein (I-FABP), and I-FABP levels in the intestines of children with severe NEC are significantly higher than those of infants who do not need surgery, suggesting that I-FABP levels might indicate the severity of the disease. The levels of an intestinal tight junction protein, claudin-3, are also elevated in children with NEC, implying a loss of intestinal wall integrity [73]. Plasma levels of the inter-α-inhibitor protein (which helps to regulate systemic inflammation) are significantly lower in children with NEC [74]. Calprotectin levels are significantly higher in the stools of children with NEC [73, 75] and correlate with disease severity [76]. Ultrasound [77] and near-infrared spectroscopy (NIRS) [78] mainly detect changes in blood flow and oxygenation, which are helpful in the early prediction of NEC. The intestinal microbiota related to the occurrence and development of NEC has also been profoundly investigated [79]. These emerging prediction methods for early diagnosis of NEC mentioned above have been systematically reviewed recently [80].

Mining potential biomarkers and integrating multi-signal scoring systems are also hot research topics. The Apolipoprotein-Serum Amyloid A (ApoSAA) scoring system, which integrates apolipoprotein-CII and a des-arginine variant of serum amyloid A, scores higher in children with NEC, and can be used to identify which children that can be taken off antibiotics[81]. Another study integrated fecal calprotectin and urinary I-FABP-binding protein to confirm the diagnosis of preterm infants with clinically suspected NEC [82]. There are also many candidate biomarkers that can be integrated and used to differentiate NEC from other benign neonatal intestinal disorders and to assess disease prognosis. In a prospective observational cohort study, determination of local intestinal oxygen saturation (rintSO2) in the urine of 27 preterm infants with the aid of NIRS, and intestinal I-FABP levels, predicted intestinal strictures after the onset of NEC, but not the rate of NEC recurrence [83]. A recent study of infants at a corrected age of 24–40 weeks [84] demonstrated higher intestinal alkaline phosphatase (IAP) and lower IAP enzyme activity in the stool of children with NEC. IAP is also a useful biomarker that responds to disease severity and could be used to improve clinical NPO duration [84].

The above are the most promising traditional biological markers available. However, an ideal biomarker would be non-invasive, specific for intestinal inflammation, and have the ability to differentiate NEC from other non-NEC diseases. It is difficult to find a single biomarker or a group of biomarkers that meet all of these criteria. Using omics technologies is possible to quantify a very large number of small molecules simultaneously with a very small number of specimens. Thus, metabolomics and proteomics have been used to explore NEC biomarkers over the last 10 years or so, and these studies have been systematically reviewed [85]. Among them, four studies dealt with metabolomics [86–89], six with proteomics [90–95], and four studies integrated metabolomics with either proteomics (one study) [96] or metagenomics (three studies) [87, 97, 98]. These published clinical studies on metabolomics and proteomics have potential to discover NEC biomarkers.

#### Nutritional support strategies

Delaying the start of enteral feeding was previously thought to reduce the incidence of NEC; however, recent studies have shown that early initiation of enteral nutrition does not increase the incidence of NEC [99, 100].

Breast milk is the only factor that has consistently been shown to improve NEC [101, 102]. The incidence of NEC in formula-only fed infants is 6–10 times higher than that in exclusively breastfed infants [103]. Recognized nutrients aside, some non-nutrient components of breast milk contribute to gastrointestinal immune function and mucosal integrity [104], including IgA, growth hormones (epidermal growth factor, insulin, and insulin-like growth factor), polyunsaturated fatty acids, and oligosaccharides. It was found that IgA in the first month of life was mainly derived from breast milk, while children with NEC at the same age had more IgA-unconjugated bacteria. Animal studies have also shown that mouse pups fed by IgA-deficient mothers are more susceptible to NEC [105]. Oligosaccharides in breast milk are known to stimulate beneficial bacteria and downregulate bacteria-associated inflammatory signaling in mouse models [106]. Breast milk-derived exosomes significantly reduced the incidence and severity of NEC in experimental animals [107] and reduced inflammation in intestinal-like organs [108]. Moreover, exosomes of colostrum origin are more protective [109], and breast milk exosomes from mothers of preterm infants enhance the proliferation and migration of IECs compared with those of full-term infants [110]. Mechanistically, breast milk-derived exosomes attenuate intestinal epithelial cell death [111] and protect intestinal stem cells from oxidative stress damage through the Wnt/β-catenin signaling pathway [112].

When maternal the breast milk supply is inadequate, infants often require supplemental donor breast milk or formula. Donor breast milk reduces the risk of NEC by 79% compared with cow's milk and other formulas [44, 113, 114]. A review of 12 clinical trials found that children fed using formula milk exhibited a higher risk of NEC [115]. The incidence of NEC was also higher when cow's milk was used [44, 116]. This is due to the protective factors contained in donor breast milk. Of course, the high reactivity of the intestine to milk proteins also contributes to the development of NEC. Studies have found that cytokines (interferon-gamma, IL-4, and IL-5) are more sensitive to milk proteins (lactoglobulin and casein) and are more prone to inflammation [117]. However, purification of milk to obtain exosomes improves NEC by stimulating cupped cells to attenuate mucin 2 (MUC2) and glucose-regulated protein 94 (GRP94) levels, which benefits children at high risk of NEC who do not have access to donor breast milk [118].

In addition, PN is commonly used in children undergoing NPO after the diagnosis of NEC. Intravenous nutrients (carbohydrates, amino acids, lipids, electrolytes, minerals and vitamins) are administered to maintain nutrition while resting the intestine. PN starts with adequate protein (3.5–4 g/kg/day) to maintain a positive nitrogen balance, improve body weight, and repair damaged tissues [10, 119–121]. However, some studies have shown that PN did not significantly improve the prognosis of NEC, nor did it reduce the proportion of children undergoing surgery or hospital mortality [122]; thus, the role of PN needs to be further confirmed.

#### New drug development

Novel biological agents might play a role in the treatment of NEC. Heparin-conjugated epidermal growth factor (EGF)-like growth factor reversed NEC in multiple animal models by promoting mucosal healing [123], restoring intestinal stem cell function [124], and improving microcirculation [57, 125]. A readily absorbed, nontoxic oligosaccharide, C34, is a 2-acetamidopyranoside (MW 389) with the formula C_17_H_27_NO_9_, which attenuated intestinal inflammation by inhibiting TLR4 signaling, ameliorated NEC lesions in mice and piglets, and reduced inflammation in human intestinal organoids obtained during NEC treatment [126]. Breast milk oligosaccharides have also been shown to have an important role in NEC treatment [127, 128]. Amniotic fluid is rich in growth factors to protect the mucosa and inhibits TLR4 signaling to reduce inflammation; thus, the use of simulated amniotic fluid is beneficial in the treatment of NEC [53, 129, 130]. Oral lactoferrin (with or without prebiotics) has been shown to be effective in blocking the development of NEC [131]. These therapies still need to be refined in clinical trials before they can be widely used in the clinical setting. The current hot topic is the treatment of NEC with various stem cells (bone marrow mesenchymal stem cells, amniotic fluid stem cells, and umbilical cord blood stem cells) and their exosomes, in addition to the treatment of NEC using breast milk exosomes mentioned above [132]. Recent studies have also identified some new therapeutic pathways and targets. Silencing angiopoietin-2 to block the Notch signaling pathway reduces lipopolysaccharide-induced inflammation, barrier dysfunction, and endoplasmic reticulum stress in intestinal epithelial cells [133]. The regulatory role of IL1-β on intestinal epithelial cell tight junctions and potential targeted therapies has also been studied [134]. The low-dose cyclooxygenase 2 (COX-2) inhibitor, celecoxib, improved the histopathological profile of the ileum of NEC rats, attenuated oxidative stress and inflammation, and reduced epithelial cell apoptosis, making it a potential therapeutic approach for NEC [135]. Sodium butyrate alleviated intestinal inflammation in mice with necrotizing small intestinal colitis [136]. Intestinal supplementation with dihydroxyacetone (DHA) might reduce the incidence of NEC in preterm infants by modulating the production of regulatory cytokines through its immunomodulatory effects [137]. Fecal filtrate transplantation (FFT) is effective in preventing NEC with no significant side effects [138] and amniotic fluid stem cell injection therapy prevented epithelial cell damage in necrotizing small bowel colitis [139]. Finally, tocilizumab inhibition of IL-6 may be a potential option for the treatment of NEC [140].

### Treatment of moderate NEC

#### Intravenous fluids, respiratory support, and condition monitoring

Supportive infusions include standard metabolic and hydride resuscitation (electrolyte and glucose added solutions) [141] to avoid water overload or excessive positive balance. A meta-analysis found that fluid restriction significantly reduced the incidence of NEC [142]. Preterm infants can develop NEC 48 h after receiving a transfusion [143, 144]; therefore, for children at risk for NEC, the potential risk of fluid overload should be considered [145]. Neurological, cardiac, hepatic, renal, and pulmonary organ function need to be evaluated regularly as NEC progresses [145]. Blood gas and the metabolic/electrolyte balance are checked regularly, different levels of respiratory support (from oxygen delivery to mechanical assistance) are provided [146, 147], and children with progressive disease need to be treated in intensive care and monitored for indications for surgery [145].

#### Antibiotic therapy

Standardized antibiotic therapy for NEC aims to provide a broad-spectrum combination of antibiotics targeting gram-negative, gram-positive, and anaerobic bacteria [141]. The collection of various body fluid samples (blood, oral swabs, stool, urine, ascites, or cerebrospinal fluid) for bacteriological culture is initiated at the Bell I stage. The most common bacteria in confirmed cases of NEC include *Escherichia coli*, *Enterobacter*, *Klebsiella*, and Coagulase-negative Staphylococci [146]. The classical antibiotic regimen combines gentamicin and ampicillin, in combination with metronidazole (Europe) or clindamycin (USA) for 10–14 days of treatment [128]. However, there are no prospective or randomized studies of the safety or efficacy of the latter two antibiotics, and there has been only one case report of intestinal stricture that occurred in a child following their use [148]. Other antibiotic regimens may be used to target specific bacteria or resistance, for example, vancomycin is used to treat increasingly common Coagulase-negative Staphylococci infections; third-generation cephalosporins may be added in the treatment of children with renal failure and severe NEC. The most recent recommendations from the Infectious Diseases Society of America (IDSA) and the Surgical Infection Society include one of the following regimens: ampicillin, gentamicin, and metronidazole; or ampicillin, cefotaxime, and metronidazole or meropenem. It is also recommended that 7–10 days of treatment after etiological control is achieved is sufficient [149, 150]. Considering the scope of the disease and the potential need for surgical intervention, neonatologists, infectious disease specialists, surgeons, and pharmacists should collaborate to develop antibiotic regimens for NEC. Antifungal and viral therapy for children with NEC should be individually designed [151].

### Treatment of severe NEC

#### Controversies in the surgical treatment of severe NEC

For children with mild symptoms, conservative treatment can be used to alleviate the symptoms, while surgical management may be necessary for children with severe symptoms. Pneumoperitoneum and clinical deterioration remain the most common indications for operative treatment [152]. However, the criteria for determining surgical indications have not been fully unified, and different doctors may have different judgment results. In addition, there are certain controversies in terms of surgical timing, surgical methods, and postoperative care.

Surgery timing: The time to perform a surgical intervention in necrotizing enterocolitis remains a challenge for the pediatric surgeon. There is the article reporting that by comprehensively analyzing the risk factors of conservative treatment and surgical treatment through imaging findings to predict the timing of surgery, the results show that when ultrasound examination reveals thickening of the intestinal wall and poor peristalsis, early surgery is recommended [153]. There is the study applied multivariate logistic regression modeling to identify factors that could provide accurate risk of surgical NEC. Children requiring surgical treatment have presented an antecedent of respiratory distress (worsening of the ventilatory requirements) in the perinatal period, they present higher values of glycemia at diagnosis of the illness, debut with coagulopathy and have in laboratory findings marked neutrophilia [154]. The choice of surgical timing needs to be weighed and judged according to the specific situation of the child.

Surgical methods: Current surgical strategies for necrotizing enterocolitis (NEC) include primary drainage, resection with enterostomies, and primary anastomosis. The research shows, the postoperative outcomes in newborns undergoing laparotomy were associated with the surgical type, which was determined by disease location in the bowel [155]. There are also different practices in how to avoid damage to the intestinal wall and how to deal with residuals in the intestinal cavity during surgery. Necrotizing enterocolitis (NEC) often is associated with extensive bowel necrosis. These cases may require extensive enterectomy and the formation of high or multiple stomas, and frequently are complicated by short bowel syndrome, excessive fluid losses, fistulas, stenosis, and skin breakdown. A report describes a “clip and drop-back” technique, followed by delayed anastomosis performed 48–72 h later. The technique is a useful addition to the pediatric surgeon’s operative armamentarium in selective cases [156]. Some doctors advocate enteroplasty, and late abdominal resurgery was performed after necrotizing enterocolitis and spontaneous bowel perforation. Late abdominal reoperations occurred in 15% of patients with NEC with nil mortality [157].

Postoperative care: As for the specific measures of postoperative care, some doctors advocate giving patients antibiotics, nutritional support and other treatments. But unlike most surgical diseases, clear guidelines for the type and duration of peri-operative antibiotic therapy have not been established. There is a high degree of variability in the antibiotic regimen for the treatment of NEC, even within a single NICU, with no regimen appearing superior over another. This research highlights the need for guidelines in the antibiotic treatment of NEC and suggests that an abbreviated course of post-operative antibiotics may be safe [158]. Meanwhile, both physiological and psychological responses need to be understood in light of family centered care [159].

#### Nutritional management of short bowel syndrome (parenteral and enteral nutrition)

Short bowel syndrome (SBS) is the most serious complication in children with NEC. Prolonged ischemic necrosis of the intestine requires excessive resection to remove the necrotic tissue, leading to functional failure along with ultra-short length of the residual intestine and structural abnormalities [160]. Neonates who develop SBS require long-term PN support and are prone to nutritional deficiencies, leading to poor growth and development [17], and are at risk of developing neurodevelopmental disorders. Individualized treatment plans and surgical treatment with limited bowel resection are the basis for avoiding the eventual development of severe NEC into SBS [14]. The basic principle of providing optimal nutritional support for children is to stimulate the residual intestine as much as possible, while ensuring the energy needed for growth and maintaining the normal growth and function of the residual intestine. This requires a combination of PN and EN and the treatment plan needs to be frequently evaluated and revised to adapt to the growth and intestinal needs of the child, developing "intestinal rehabilitation" and rejecting intestinal transplantation [160].

#### Intervention studies for neurodevelopmental disorders

NEC is not only an intestinal disorder [161, 162], but also its broader sequelae include systemic inflammation, hypoxia, ischemia and, in severe cases, triggered multisystem organ dysfunction, particularly affecting brain and lung function [163]. NEC has been reviewed as an independent risk factor for neurodevelopmental delays and poor neurocognition in preterm infants [164–166]. Children exhibit neurological and motor dysplasia around 2 years of age, and cognitive deficits can persist into school age [164, 167, 168]. The pathogenesis might be related to the NEC-associated microbiome alterations to neurotransmitter levels in the brain development of affected children [169]. Murine models have demonstrated an irreversible increase in blood–brain barrier (BBB) permeability and barrier dysfunction caused by altered bacterial composition affecting tight junction proteins, resulting in reduced expression of short-chain fatty acids (SCFAs) in the brain [164]. Immune cells, such as microglia and astrocytes, maintain normal brain function; participate in synaptic pruning, formation and transmission; and regulate neurogenesis, neuronal migration, and synaptic plasticity [170]. As the severity of NEC disease increases, protective factors are exposed to an inflammatory environment and the disease progresses into surgical NEC [171]. Increased levels of pro-inflammatory factors [161] lead to poorer neurodevelopmental outcomes in affected children.

The TLR4 signaling pathway is now known to be involved in NEC-related encephalopathy [172]. In mouse models of NEC, TLR4 activation activates its endogenous ligand, high mobility base box 1 (HMGB1), and is released from the intestine, activating TLR4 on microglia, leading to reactive oxygen species (ROS) accumulation in the brain, oligodendrocyte progenitor cells (OPCs) loss, myelin disorders, and cognitive impairment. Anti-inflammatory and antioxidant treatments targeting microglia alleviate neurological dysfunction and thus represent new therapeutic targets for NEC-related brain injury. Recent studies have found that CD4^+^ T cells in the intestine of patients with NEC and NEC mice can infiltrate into the brain and secrete IFN-γ, which induces microglia activation and myelin loss, leading to brain injury, suggesting that early management of intestinal inflammation in children with NEC can improve the neurological prognosis of NEC [173].

#### Remission of pulmonary complications

Pulmonary injury, characterized by neutrophil infiltration and upregulation of inflammatory factors, occurs in approximately 15% of children with NEC. Recent studies on NEC-induced lung injury have revealed a TLR4-mediated pathogenesis similar to that of NEC-associated brain injury [174, 175]. TLR4 is highly expressed in the lung epithelium of NEC mice and can be activated by HMGB1 from the intestinal epithelium to upregulate C–X–C motif chemokine ligand 5 (CXCL5) recruitment of neutrophils. A novel TLR4 small molecule inhibitor, C34, reverses this inflammatory cascade and prevents NEC from triggering lung injury [174]. Th17 in NEC can drive cytokine upregulation and immune cell infiltration leading to inflammation and lung injury [176, 177]. Upregulation of the chemokine C–C motif chemokine ligand 25 (CCL25) mediates the development of inflammation, and TLR4 in the lung activates downstream CCL25 upregulation, causing protective T cell (regulatory T cells (Tregs)) depletion in the lung epithelium [175]. A recent study has shown that a "PDF system" (pre-digested fat system), in which a nutritional formula rich in pre-digested glycerol (without lipase action) was given in a mouse model. The PDF system, which affects lung maturation and reduces intrapulmonary ROS, protects against NEC-induced lung injury, and increases lung maturation in non-NEC mice, can significantly reduce the severity of NEC [178].

## Conclusions

In this review, we have systematically described the possible pathogenesis of NEC in terms of epidemiology, risk factors, pathophysiology, clinical diagnosis, and manifestations. Intervention strategies to arrest the development of NEC have been slow to be perfected because of the complexity of its etiology and pathogenesis, and the diversity of its clinical manifestations. Therefore, we categorized and summarized the disease with respect to its multifaceted pathogenesis and clinical manifestations, with preventive tools including maternal health care to avoiding preterm birth, probiotic and breastfeeding for preterm infants (including donor breast milk) to avoid formula feeding, and targeting IL-10 and TLR4 and chondroitin sulfate to avoid infection and inflammation. We also have discussed new approaches to improve ischemia and reperfusion like RIC, and therapeutic measures for suspected NEC, such as NPO, early identification biomarkers, nutritional support, and exploration of new drug therapies. For moderate to severe NEC, not only the controversies in the surgical management have been discussed, but also new targets for treating complications have been identified after symptomatic and surgical treatment. We have talked about hot topics in NEC, such as the development of new scores and clinical scenarios. We also have described current interventions and management approaches, provided a more comprehensive review of current approaches for NEC prevention and treatment, thus deepening our understanding of intervention mechanisms. Considering the irreversible suffering caused by severe NEC in children and the poor long-term prognosis caused by surgical treatment, we should focus on providing guidance for better diagnosis and prevention of the disease before the onset and at the suspected stage of NEC. The current study offers the possibility of developing more adapted individualized prevention and treatment strategies in future.

## Data Availability

The datasets used and analyzed during the current study are available from the corresponding author Yunyun Xu (Email: xyy0208@suda.edu.cn) upon reasonable request.

## Abbreviations

Apo-SAA: Apolipoprotein-Serum Amyloid A
BBB: Blood–Brain Barrier
CCL25: C–C Motif Chemokine Ligand 25
CDC: Centers for Disease Control and Prevention
COX-2: Cyclooxygenase 2
CRP: C-Reactive Protein
CS: Chondroitin Sulfate
CXCL5: C–X–C Motif Chemokine Ligand 5
DHA: Dihydroxyacetone
EGF: Epidermal Growth Factor
ELBWIs: Extremely Low Birth Weight Infants
FFT: Fecal Filtrate Transplantation
GLUT1: Glucose Transporter 1
GRP94: Glucose-Regulated Protein 94
HIF-1: Hypoxia-Inducible Factor 1
HMGB1: High Mobility Base Box 1
IAP: Intestinal Alkaline Phosphatase
IDSA: Infectious Diseases Society of America
IF: Intestinal Failure
I-FABP: Intestinal Fatty Acid Binding Protein
IL-10: Interleukin 10
INC: International Neonatal Consortium
LBW: Low Birth Weight
LBWIs: Low Birth Weight Infants
MUC2: Mucin 2
NDD: Neurodevelopmental Disorders
NEC: Necrotizing Enterocolitis
NIRS: Near-Infrared Spectroscopy
OPCs: Oligodendrocyte Progenitor Cells
PCT: Procalcitonin
PDF system: Pre-Digested Fat System
PGE2: Prostaglandin E2
PN: Parenteral Nutrition
PTGER4/EP4: Prostaglandin E Receptor 4
rintSO2: Regional Intestinal Oxygen Saturation
RIC: Remote ischemic conditioning
ROS: Reactive Oxygen Species
SBS: Short Bowel Syndrome
SCFAs: Short-Chain Fatty Acids
SFPs: Standardized Feeding Protocols
TLR4: Toll-Like Receptor 4
Tregs: Regulatory T Cells
UKNC-NEC: UK Neonatal Collaborative NEC
VLBWIs: Very Low Birth Weight Infants
VON: Vermont Oxford Network
